# Influence of Maternal Diet and Environmental Factors on Fetal Development

**DOI:** 10.3390/nu15194094

**Published:** 2023-09-22

**Authors:** Asim K. Duttaroy

**Affiliations:** Department of Nutrition, Faculty of Medicine, Institute of Basic Medical Sciences, University of Oslo, 0316 Oslo, Norway; a.k.duttaroy@medisin.uio.no

This Special Issue of Nutrients, “Influence of Maternal Diet and Environmental Factors on Fetal Development”, requests articles on the roles of maternal diet and environmental factors such as microbiota, plastics, and endocrine disruptive chemicals impact fetal development.

The maternal diet is critically important before and during pregnancy for the optimum development of the fetus [1]. The prenatal environment, such as maternal and environmental factors, can significantly influence fetal and adult life. Maternal nutrition supports adequate fetal development and reduces risks of possible health complications for the offspring and adult life [2]. The placenta is the essential organ that affects these processes by supplying maternal nutrients and environmental factors. The placenta provides the fetus with maternal nutrients, hormones, and oxygen, contributing to fetal growth and development throughout pregnancy [3]. Apart from maternal factors, several environmental factors modulate the growth and development of the fetal-placental axis via different mechanisms [4]. These maternal factors influence placental development and thereby impact fetal growth. Therefore, the nutritional and environmental factors encountered throughout pregnancy can influence placental growth and function via endocrinal, epigenetic, and other pathways [5,6].

Maternal n-3 and n-6 long-chain polyunsaturated fatty acids status are essential to fetoplacental growth and development [2,7,8,9,10]. Maternal docosahexaenoic acid, 22:6n-3 (DHA) deficiency could affect fetal neurodevelopment, fetoplacental changes in epigenetics, offspring growth, and lipogenic capacity [5]. Alterations in membrane phospholipid fatty acid composition can affect the function of the neurons by changing the membrane receptors, ion channels, enzymes, and fatty acid-derived second messengers. A high-fat diet during pregnancy can increase the risk of neurological behaviors later in life in offspring [11,12]. Mice studies showed that n-3 fatty acid deficiency during fetal development impacted adipose browning and postnatal musculoskeletal development of the offspring [13]; however, no such information is available in humans. Adipose tissue is a target for signals of nutrients, hormones, and epigenetics that regulate fetal growth and development [14,15]. Therefore, more information is required on whether disruptions in the maternal nutritional, hormonal, epigenetic, and gut microbiota in obesity alter fetal growth and later-life adiposity [14].

Various environmental chemicals, including endocrine-disrupting chemicals (EDCs), can alter the cellular differentiation process and epigenome during the developmental stages in the fetoplacental axis [16]. Exposure to EDCs in the early developmental stage can also induce metabolic diseases [16,17]. More information is required on the mechanisms of action of EDCs that lead to diseases later in life. An important role of epigenetic mechanisms in the effects of EDCs and other environmental chemicals was reported [16,17,18,19]. Bisphenols can affect placental development and fetal programming, but the mechanisms are yet to be established [20]. Pesticide exposure, including organochlorine and organophosphorus pesticides, and a risk of fetal development or pre-term birth are known. Yet more studies are still needed with larger sample sizes, careful considerations of confounders, and accuracy of outcome measurements. Changes in endocrine status may alter the physiology and metabolism of the developing fetus and contribute to several diseases in adult life. 

During pregnancy, the composition of the maternal gut microbiota and changes have significant consequences for fetal development and adult health [12]. Gut microbiota is involved in fetal growth and development [12] and regulates pre-eclampsia by producing short-chain fatty acids [18]. Gut microbiota also affects offspring’s metabolism and immune system. Many factors, such as maternal diet, BMI, metabolic disorders, ethnicity, and geographic and environmental factors, modulate the maternal–fetal microbiota [21]. A healthy maternal diet is required to maintain healthy gut microbiota, contributing to the fetus’s and newborn’s intestinal microbiota. Prenatal use of pro- and prebiotic treatment on offspring’s health is known, although detailed studies regarding the type, dosage, and timing of their intake during pregnancy are required. Despite the convincing results, some critical points and significant evidence still need to be included. 

The maternal nutritional and environmental factors can alter the epigenetic state of the fetal genome and imprint gene expression [22]. Epigenetic alterations related to maternal nutrition and environmental exposures may affect fetal growth [23]. Further information is required linking maternal nutritional and environmental cues to fetoplacental pathology, with consequences for fetal development and adult life.

This Special Issue invites articles on maternal nutrients, environmental factors, and their impacts on fetal and neonatal development and adult health. In addition, articles are invited on the maternal dietary factors and other potential modulators of the maternal–fetal microbiota axis during pregnancy, impacting offspring’s microbiota and health.
